# The integrated GOR-COVID-19 health monitor: research-informed policy-making through dialogue

**DOI:** 10.1093/eurpub/ckac129.483

**Published:** 2022-10-25

**Authors:** N Jansen, E Marra, M de Vetten-Mc Mahon, M Dückers

**Affiliations:** 1 Impact, ARQ National Psychotrauma Centre, Diemen, Netherlands; 2 Centre for Environmental Safety and Security, RIVM, Bilthoven, Netherlands; 3 Disasters and Environmental Hazards, NIVEL, Utrecht, Netherlands; 4 Faculty of Behavioural and Social Sciences, University of Groningen, Groningen, Netherlands

## Abstract

**Introduction:**

Like in many other countries, the COVID-19 pandemic and the government restrictions introduced to contain the spread of the virus had major consequences for the health and wellbeing of the population in the Netherlands. To monitor the short and long-term public health impact, a nationally coordinated research program was initiated with the intention to guide decision-making by local and national public health authorities. This contribution presents the process to establish a continuous dialogue with end-users of information to add focus to the monitor, make sense of the findings and formulate policy recommendation and practical guidance, both at the national and regional level.

**Methods:**

To facilitate the translation and dissemination of research results among policy makers, practitioners and scientists, an ongoing series of dialogue sessions is organized during the monitoring program. Apart from the objective of evidence-informed public health decision-making, the dialectic process seeks to ensure multi-sectoral learning and co-creation and contribute to a broad sense of ownership among stakeholders. National and regional health participants serve as hub coordinators. New stakeholders are invited and will be actively approached wherever considered relevant.

**Results:**

At the EUPHA conference experiences with organizing the dialogue in app. the first two years of the program will be presented together with preliminary results and a reflection on factors that helped or hindered the implementation and uptake of findings.

**Discussion:**

Monitoring data collected using robust methods and analyzed in such a way that vulnerability factors are carefully considered, is invaluable for decision-making. However, in order to effectively serve as guidance to public health policy, whether in the context of the COVID-19 pandemic or in non-crisis situations, a constructive, ongoing exchange between end-users of the information needs to be facilitated.

